# Recombination Is Responsible for the Increased Recovery of Drug-Resistant Mutants with Hypermutated Genomes in Resting Yeast Diploids Expressing APOBEC Deaminases

**DOI:** 10.3389/fgene.2017.00202

**Published:** 2017-12-12

**Authors:** Artem G. Lada, Elena I. Stepchenkova, Anna S. Zhuk, Sergei F. Kliver, Igor B. Rogozin, Dmitrii E. Polev, Alok Dhar, Youri I. Pavlov

**Affiliations:** ^1^Eppley Institute for Research in Cancer and Allied Diseases, University of Nebraska Medical Center, Omaha, NE, United States; ^2^Department of Microbiology and Molecular Genetics, University of California, Davis, Davis, CA, United States; ^3^Department of Genetics and Biotechnology, Saint Petersburg State University, Saint Petersburg, Russia; ^4^Vavilov Institute of General Genetics, Russian Academy of Sciences, Saint Petersburg, Russia; ^5^National Center for Biotechnology Information, National Library of Medicine, National Institutes of Health, Bethesda, MD, United States; ^6^Institute of Cytology and Genetics, Novosibirsk, Russia; ^7^Research Resource Center “Biobank”, Research Park, Saint-Petersburg State University, Saint Petersburg, Russia; ^8^Department of Genetics, Cell Biology and Anatomy and Vice Chancellor of Research Core, University of Nebraska Medical Center, Omaha, NE, United States; ^9^Department of Biochemistry and Molecular Biology, University of Nebraska Medical Center, Omaha, NE, United States; ^10^Department of Pathology and Microbiology, University of Nebraska Medical Center, Omaha, NE, United States; ^11^Department of Genetics Cell Biology and Anatomy, University of Nebraska Medical Center, Omaha, NE, United States

**Keywords:** APOBECs, kataegis, mutants in resting diploid yeast, recombination, next generation sequencing, tumorigenesis

## Abstract

DNA editing deaminases (APOBECs) are implicated in generation of mutations in somatic cells during tumorigenesis. APOBEC-dependent mutagenesis is thought to occur during transient exposure of unprotected single-stranded DNA. Mutations frequently occur in clusters (*kataegis*). We investigated mechanisms of mutant generation in growing and resting diploid yeast expressing APOBEC from sea lamprey, PmCDA1, whose kataegistic effect was previously shown to be associated with transcription. We have found that the frequency of canavanine-resistant mutants kept raising after growth cessation, while the profile of transcription remained unchanged. Surprisingly, the overall number of mutations in the genomes did not elevate in resting cells. Thus, mutations were accumulated during vigorous growth stage with both intense replication and transcription. We found that the elevated recovery of *can1* mutant clones in non-growing cells is the result of loss of heterozygosity (LOH) leading to clusters of homozygous mutations in the chromosomal regions distal to the reporter gene. We confirmed that recombination frequency in resting cells was elevated by orders of magnitude, suggesting that cells were transiently committed to meiotic levels of recombination, a process referred to in yeast genetics as return-to-growth. In its extreme, on day 6 of starvation, a few mutant clones were haploid, likely resulting from completed meiosis. Distribution of mutations along chromosomes indicated that PmCDA1 was active during ongoing recombination events and sometimes produced characteristic *kataegis* near initial breakpoints. AID and APOBEC1 behaved similar to PmCDA1. We conclude that replication, transcription, and mitotic recombination contribute to the recovered APOBEC-induced mutations in resting diploids. The mechanism is relevant to the initial stages of oncogenic transformation in terminally differentiated cells, when recombination may lead to the LOH exposing recessive mutations induced by APOBECs in cell’s history and to acquisition of new mutations near original break.

## Introduction

Cytosine RNA/DNA editing deaminases of the APOBEC family are prominent intrinsic mutagens imminent for healthy immunity, but when regulation of their precise targeting is impaired, they become prominent risk factor in tumorigenesis (Neuberger et al., 2003; Burns et al., 2013b; Roberts and Gordenin, 2014a). Hypermutability is connected to etiology of tumors (Loeb, 2001). In many tumors, deaminases genes are overexpressed and genomes of tumors harbor mutations with clear APOBEC signatures (Burns et al., 2013a; Rebhandl et al., 2015). An additional feature of mutation profiles of cancer genomes is clustering of changes in DNA, “*kataegis*” (Nik-Zainal et al., 2012, 2014; Roberts et al., 2012). Because deaminases possess an ability to act processively on single-stranded DNA (ssDNA) (Pham et al., 2003), *kataegis* by APOBECs is explained by the transient appearance of unprotected ssDNA during recombination, replication, or transcription (Larson and Maizels, 2004; Taylor et al., 2013; Chan and Gordenin, 2015; Lada et al., 2015; Saini et al., 2017), creating short-lived hypermutable state without the loss of fitness (Roberts and Gordenin, 2014b). In relevance to the origin of cancer, which is frequently initiated in dormant cells, it is imperative to understand the mechanisms of deaminase-induced mutagenesis in resting versus actively replicating cells.

The mechanisms of mutagenesis are relatively well explored in dividing cells of microorganisms. Most of the mutations arising in proliferating cells are induced in a replication-dependent manner (Mertz et al., 2017). Errors of replicative polymerases *per se*, instigated by aberrations in DNA synthesis precursor pools, genetic variants of polymerases with low fidelity, or combination of these factors, as well as failures of replication-coupled mismatch repair, contribute to spontaneous mutagenesis (reviewed in Pavlov et al., 2006; Arana and Kunkel, 2010; Waisertreiger et al., 2012; Hoopes et al., 2016). Additionally, damage of DNA by exogenous and endogenous agents leads to miscoding lesions or stalls replication that promotes the recruitment of error-prone translesion synthesis (TLS) DNA polymerases (Goodman and Woodgate, 2013). The fixation of mutation by TLS on damaged template occurs during replication in the S-phase or later, in a post-replicative manner, in the G2 phase (Quinet et al., 2014).

The processes underlying mutagenesis in non-proliferative cells are not as fully understood. Induction of mutations in non-dividing cells, e.g., microorganisms during starvation, terminally differentiated somatic cells, or dormant stem cells can have serious consequences for cell’s fate. The so-called “adaptive” mutations in bacteria and yeast are induced predominantly in non-dividing cells (reviewed in Rosenberg, 2001). Their appearance is connected to error-prone DNA pols (Maisnier-Patin and Roth, 2015). The fixation of mutations induced by UV in stationary yeast cells occurs before the cells enter the S-phase when nucleotide excision repair-generated gaps in the DNA of non-dividing cells are filled by TLS polymerases (Kozmin and Jinks-Robertson, 2013). Thus, the repair processes in resting cells can contribute to the origin of mutations.

**FIGURE 1:**
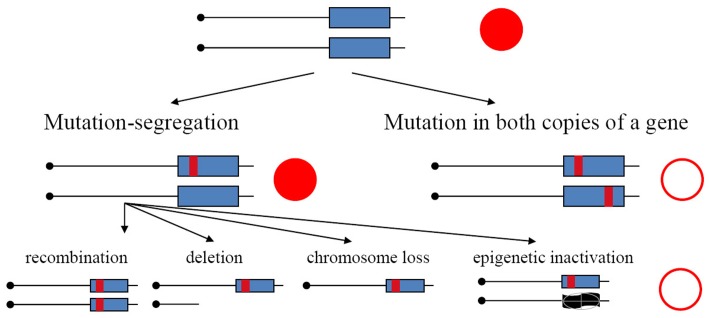
Mechanisms of the generation of recessive mutants in diploids. Mutation-segregation is a two-step process when a wild copy of the gene is functionally or physically absent. Simultaneous mutation in two copies immediately produces a mutant clone but it is a rare event. Red filled circle—wild-type phenotype; open circle—mutant phenotype.

In the current study, we have used yeast diploids as a model of mutation processes occurring in resting somatic cells. We have found previously that APOBECs expressed in yeast induce substantially more mutations in the genomes of diploids than haploids (Lada et al., 2012, 2013), indicating that in diploids we recovered descendants of transiently hypermutable cells (Lada et al., 2013). Main arguments in favor of this explanation are as follows. Mutations in haploids and diploids have different consequences. They immediately result in a phenotypic change in haploids but, in the heterozygous state, they will be detectable in diploids only in the rare cases when they are dominant. Mutants in diploid tumors with recessive mutation in both copies of the gene arise by two-hit process (Knudson, 1971). It was established in classical studies that mutants in diploids arising spontaneously, or induced by UV light or X-rays, appear by a two-step mechanism: recessive mutation in one homolog and loss of heterozygosity (LOH) (Gordenin and Inge-Vechtomov, 1981; Chernov et al., 1985) (**Figure 1**, left branch). This happen because, typically, mutagens introduce damage in DNA and mutations arise during the repair/bypass of this damage. Such repair events require DNA breakage as an intermediate. Therefore, most mutagens induce, in addition to mutations, an acute increase of mitotic recombination or chromosome loss events. Some conditions, however, for example, increases of error rates during inaccurate replication, are highly mutagenic but do not induce recombination. In such a case, mutants in diploids should be extremely rare, because independent mutations in both homologous genes are needed (**Figure 1**, right branch), which theoretically is a square of mutant frequency in haploids; however, mutants unexpectedly occur at a frequency far exceeding these expectations (Pavlov et al., 1988, 1991; Noskov et al., 1990; Tran et al., 1999; Lada et al., 2013; Kane and Shcherbakova, 2014). The genomes of these drug-resistant mutants accumulate much more mutations induced by the replicative mutagen base analog 6-*N*-hydroxylaminopurine, whose deoxynucleoside triphosphate form taint DNA synthesis precursor pools, or by editing deaminases, than haploids (Lada et al., 2012, 2013; Taylor et al., 2014). Higher levels of accumulation of mutations in genomes of diploids versus haploids are also seen during inaccurate replication in DNA polymerases mutants (Lujan et al., 2014). The effect brings upfront the concept of a transiently hypermutable fraction of cells (Pavlov et al., 1988; Tran et al., 1999; Lada et al., 2013). If a fraction of cells has transient spikes of mutagenesis, when the mutation rate increases beyond the rate compatible with life in haploids, then the appearance of mutants could occur with reasonable frequency in diploids, because lethal mutations are in a heterozygous state and descendants of hypermutable cells will live. The mutation rate compatible with life could be much higher in diploids. A recent study in yeast confirmed that individual cells harboring inaccurate DNA polymerase differ in mutability (Kennedy et al., 2015).

In the current study, we constantly expressed APOBEC deaminases in yeast diploid cultures, that rapidly reached stationary phase of growth and stayed dormant for a long time. We observed the sharp increase in yield of drug-resistant mutant clones but the overall number of induced mutations per genome remained constant, suggesting that most of the *kataegistic* cluster-prone mutations induced by deamination occurred during the active phase of growth. We found that the increase of the frequency of the drug-resistant mutants was due to recombination between *CAN1* gene (or *URA3* gene, depending on the type of selection for drug-resistance) and centromere during the continuation of the stationary phase. Thus, starvation-induced recombination contributes to the recovery of heavily mutated clones with kataegistic patches in non-dividing diploid cells whose genomic cytosines have been deaminated by APOBECs. In addition, some mutation clusters were induced by action of APOBECs during the recombination process itself. The results reveal one of the mechanisms of phenotypic changes in non-dividing cells that can be relevant for the initiation of cancer in terminally differentiated cells.

## Materials and Methods

### Plasmids

The pESC-TRP-HXT13::ADE2 was made as follows. First, we cloned the *HXT13* gene fragment into *Sac*II–*Xho*I sites of the pESC-TRP vector using an appropriately cut PCR fragment obtained with primers

HXT13-*Sac*II 5′-ATTGCCCGCGGCCTTTAACTCCTGGTTTACGG and
HXT13-*Xho*I 5′-ATTGCCTCGAGAATCGAGGATCCTATGGCACC

on the genomic DNA of yeast strain BY4742. Then, we cloned the fragment of the *ADE2* gene into the *Nco*I–*Bcl*I sites of the *HXT13* region of pESC-TRP-HXT13, using a PCR fragment amplified on the DNA of the LAN201-uraΔ strain (Stepchenkova et al., 2017) with primers:

ADE2-*Nco*I 5′-ATTGCCCATGGAACTTGACTAGCGCACTACC
ADE2-*Bcl*I 5′-ATTGCTGATCACACTGGAATCGAATGACGC.

Plasmids for galactose-inducible expressing of APOBEC deaminases (pESC-LEU-PmCDA1, pESC-LEU-AID, and pESC-LEU-APOBEC1 has been described previously (Lada et al., 2011, 2015).

### Yeast Strains

The basic LAN201 strain whose genome is sequenced (Lada et al., 2013), is CG379 background (Morrison et al., 1993; Shcherbakova and Kunkel, 1999; Young and Court, 2008), which is in its turn close to S288C (Mortimer and Johnston, 1986). For the experiments with a prolonged induction of deaminases, we have used the following strains: LAN200 [the genome used here as a reference in NGS data analysis; isogenic for LAN201, except for the disruption of the *UNG1* gene and a few SNVs (Lada et al., 2013)], LAN210 (HO-induced diploid of LAN200), and LAN211 (HO-induced diploid of LAN201). For recombination studies, we constructed a new strain, ES20 (*MAT*a*/MATα ade2::kanMX/ade2::kanMX hxt13::ADE2/HXT13 URA3/ura3Δ ung1::hphMX/ung1::hphMX CAN1/can1-G1018A lys2-Tn5-13/lys2-Tn5-13 trp1-289/trp1-289 his7-2/his7-2 leu2-3,112/leu2-3,112*), allowing for the selection of genetic events leading to the loss of the wild-type *CAN1* gene and classification of the types of events in the left arm of chromosome V as described in the section “Results”. As a starting material for the construction of ES20 we used LAN201-ura3Δ, a derivative of LAN201 with a deletion of the *URA3* gene made as described in (Stepchenkova et al. 2017). Next, we created a T1 strain (*MATa ura3Δ lys2-*Tn*5-13 trp1-289 his7-2 leu2-3,112*) by converting the *ade5-1* allele in LAN201-ura3Δ to the wild-type *ADE5* by transformation with wild-type *ADE5,7* PCR product obtained with primers amplifying a 802 bp part of *ADE5,7* gene covering the site mutated in *ade5-1* (C1158A), on a DNA template from the strain BY4742 (*ADE5,7*-890 5′-GTTAGAATATAATGTCAGATTCGG and *ADE5,7*-1668R 5′-GAATGTCAAGAGCACCAGTGGC). Strain T1-ade2 (T1, but *ade2::kanMX*) was constructed from T1 by transformation with the DNA of *ade2::kanMX* PCR cassette amplified using chromosomal DNA of *ade2::kanMX* mutant available from the yeast BY4742 deletion library (Invitrogen) with primers ADE2-F 5′-AACTTGACTAGCGCACTACC and ADE2-R 5′-CACTGGAATCGAATGACGC. The T1-derivative with *ung1::hphMX* mutation, T1-ung1, was obtained by transformation with the *ung1::hphMX* PCR product followed by hygromycin B selection. We obtained this PCR fragment using primers: UNG1-F 5′-TGGAGTCGTGACCATTCTACCTAC and UNG1-R 5′-GCTCCAGTGTTCACTTTACTGAACG) on the genomic DNA template from LAN200 strain (Lada et al., 2011). T1-ade2 ung1 was used to generate parent strains of the opposite mating type for further ES20 diploid construction.

#### *MAT***a** Parent Lineage

The *URA3* derivative of the T1-ade2-ung1 strain was made by the transformation of the parental strain with the PCR fragment carrying the wild-type allele (we amplified the chromosomal region flanking the *URA3* gene of the LAN201 strain using primers *URA3*-F 5′-AAGAAGAGTATTGAGAAGGG and *URA3*-R 5′-CCTACACGTTCGCTATGC). A variant of this strain with the insertion of the *ADE2* gene in chromosome V (*MAT***a** *lys2-Tn5-13 trp1-289 his7-2 leu2-3,112 ade2::kanMX ung1::hphMX hxt13::ADE2*) was obtained by transformation with *Sac*II–*Xho*I digested pESC-TRP-HXT13::ADE2 plasmid.

#### *MAT*α Parent Lineage

Strain T1-ade2-ung1 was made diploid by transformation with replicative plasmid pLEU-HO encoding for HO endonuclease. The diploid strain was passaged three times on YPDAU media. Clones that lost the plasmid were selected and placed on sporulation medium. After tetrad dissection, we isolated a clone isogenic to the starting strain but with the opposite mating type, T1-ade2-ung1-alpha (*MATα lys2-Tn5-13 trp1-289 his7-2 leu2-3,112 ura3Δ ade2::kanMX ung1::hphMX*). Next, we selected a spontaneous *can1* mutant and found that it has a G1018A substitution in the *CAN1*.

The final strains from the two lineages were crossed to provide the diploid ES20.

### Determination of Frequencies of Drug-Resistant Colonies Induced by Deaminases in Non-dividing Cells and Selection of Clones for Whole-Genome Sequencing

Yeast strains LAN200, LAN210, and LAN211 were transformed with plasmids expressing APOBEC deaminases (pESC-LEU-PmCDA1, pESC-LEU-AID, pESC-LEU-APOBEC1; Lada et al., 2015). Transformants were inoculated in 5 ml of synthetic complete (SC) media containing glucose and without leucine and incubated overnight with shaking at 30°C. Then, the yeast was spun down, washed with sterile water, and resuspended in 12 ml of SC media lacking leucine and glucose, but with the addition of 1% raffinose and 2% galactose. These cultures were incubated for 6 days with shaking at 30°C. Every day, a sample from these cultures has been taken and plated on SC plates with canavanine (and, in some experiments, also to the SC plates with 5-FOA), and to the SC plates lacking canavanine/5-FOA, to score resistant colonies and viability, respectively. This was done from day 0 (samples taken immediately after resuspending cells in galactose-containing media) to day 6 (the final day of the experiment). In addition, optical densities (OD_600_) of the cultures have been monitored on a daily basis using NanoDrop spectrophotometer.

One clone per a drug-containing plate from a characteristic experiment that followed average pattern of mutant induction out of three consistent starvation experiments has been picked, propagated and stored in 50% glycerol at -80°C for further DNA isolation. Genomic DNA was isolated from selected clones using glass-beads disruption followed by phenol-chloroform extraction, as described (Lada et al., 2013).

Additionally, for RNA isolation, samples have been taken from the two cultures expressing PmCDA1 on day 3 and day 6 of growth (four samples total).

### Next-Generation Sequencing and Data Analysis

Whole-genome and RNA sequencing were performed using the Illumina platform, as described (Lada et al., 2015). We have sequenced the whole genomes of 66 yeast clones (see Supplementary Table 1, column “Reference”). For analysis of SNV loads and recombination events, we have combined these results with the previously obtained sequencing data (Lada et al., 2015). A summary of sequenced and analyzed clones is in Supplementary Table 1. Raw reads processing, alignment to the reference genome, and SNV call were performed generally as described (Lada et al., 2015) with the following modifications. First, the terminal Ns were removed from the reads before processing. Second, we have used updated and improved version of genome mask instead of filtration associated with clustering to improve the quality of SNV call. Third, HaplotypeCaller from new version of GATK (3.8-0) was used to call the variants. Forth, a more rigorous filtration for the strand bias artifacts (only SNVs with FS ≤ 20 were retained in final vcf files) was applied, as we discovered that most of the false-positive SNVs have shown a high strand bias. This modified pipeline was used to process all samples, including the clones sequenced and reported before (Lada et al., 2015). The final SNV results (vcf files) are in Supplementary Data Sheet1.

We observed higher numbers of false-positive calls in clones with lower overall sequence coverage. Some clones were considered homozygous based on the manual examination of alignments that confirmed false-positivity of few heterozygous SNVs in these clones. The final ^∗^.vcf files for such clones, however, has not been edited, for the sake of uniformity of the results.

RNA sequencing data was analyzed by the standard pipeline using Top Hat, with a follow-up by Cufflinks, while using our own reference genome and annotations (Lada et al., 2015). To analyze PmCDA1 expression, sequence of pESC-LEU-PmCDA1 expression plasmid was added to the reference genome file, and annotations updated accordingly.

Visualization of NGS alignments and results of SNV calls for both pipeline optimization and figure preparation were done using Geneious releases R6–R10 (Biomatters) and Integrative Genomics Viewer (IGV) 2.3 (Broad Institute). Statistical analysis and the creation of graphs were performed in GraphPad Prism v. 5 and R.

### Determination of Recombination Rates by Fluctuation Test and Analysis of Classes of Recombinants

In two independent repeats of experiment, nine cultures for each strain were grown overnight in SC medium with glucose and without leucine to select for plasmids containing the *LEU2* gene. Cells were washed and resuspended in the same medium with glucose or with raffinose plus galactose (to induce the expression of PmCDA1). The volume of medium added and the number of cells was adjusted to obtain a fourfold dilution of overnight cultures. On days 1, 3, and 6, aliquots of the appropriately diluted cultures were plated on complete minimal plates, with or without canavanine, to estimate the number of resistant cells and live cells, respectively. Seventy colonies from each canavanine plate were tested on diagnostic media to find the ratios of different classes of recombinants/mutants in each culture. Then proportion of each type of event in the sum of data for nine cultures (560 colonies total) was calculated.

### Data Availability

All strains and plasmids are available upon request. Raw data has been deposited to SRA (accession numbers: SRP056337 and SRP100653 for genomic DNA samples, and SRP056371 for RNA-Seq data). Results of the SNV call are presented in Supplementary Data Sheet 1 (archive with vcf files).

## Results

### Frequency of Can^r^ Clones Induced by PmCDA1 Increases in Non-dividing Diploid Cells

**FIGURE 2:**
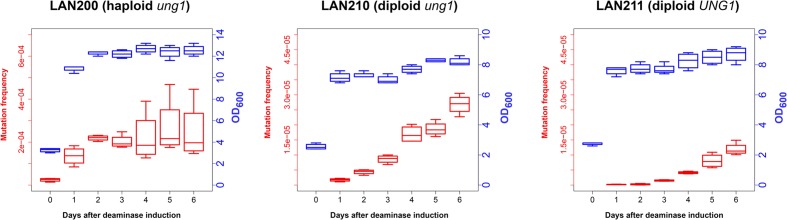
Frequency of canavanine-resistant colonies induced by deaminase in diploids increases in non-growing cultures. Mutation frequencies, red box-and-whiskers, left axes; optical densities (OD_600_ measured by NanoDrop spectrophotometer), blue box-and-whiskers, right axes. Boxes (25th and 75th percentiles) with the middle lane, median (50% percentile). Upper whisker, either maximum value or (Q_3 + 1.5 × IQR), whichever is smaller; similarly, lower whisker, either smallest value or (Q_1 – 1.5 × IQR), whichever is larger. Q_1 and Q_3, first and third quartiles; IQR, interquartile range. Experiments with LAN200 haploid strain transformed with empty vector showed that PmCDA1 induced higher mutant frequencies even on day 0 (medians of four independent cultures: 9.2 × 10^-6^ for empty vector versus 2.6 × 10^-5^ for PmCDA1, about a threefold increase), likely due to the leaky expression from the *GAL* promoter in the glucose-containing media. On the following days, mutation frequency in vector-only cultures remained stable.

To examine how deaminase mutagenesis depends on the growth stage of yeast cultures, we continuously induced PmCDA1 expression in three yeast strains for 1 week and examined the dependence of the frequency of Can^r^ clones in yeast on the day of culturing, and thus on the proliferation state of yeast cultures (**Figure 2**). LAN200 is a haploid strain, whereas LAN210 and LAN211 are diploids. All strains are isogenic except for the disruption of the *UNG1* gene in LAN200 and LAN210. This gene encodes for the base excision repair enzyme uracil-DNA-glycosylase (UDG), which excise uracil from DNA. Mutations induced in strains with a defect of UDG thus more closely represent the sites of initial deaminations (Harris et al., 2002; Petersen-Mahrt et al., 2002). APOBECs do not induce recombination in the *ung1* strains, because in the absence of the major pathway of uracil repair there are no accompanying DNA breaks (Di Noia and Neuberger, 2004; Poltoratsky et al., 2004; Rogozin et al., 2007).

In synthetic medium with galactose (inducer of deaminase genes in our strains), yeast haploids and diploids grow vigorously for 1 day and then stop dividing (growth curves data are represented in **Figure 2** with blue-colored boxes with whiskers). Mutant frequency in haploids is threefold higher with deaminase compared to the variant with the vector alone (9.2 × 10^-6^) at day 0, most likely because of a weak leaky expression of PmCDA1 in the medium with glucose, where the cultures were initially grown (note in the legend to **Figure 2** and red-colored boxes and whiskers in **Figure 2**), consistent with the strong mutagenic potential of PmCDA1, and even low concentration of the enzyme elicited some mutagenic effect. One day of incubation of haploids possessing PmCDA1 (vigorous growth period) led to a fivefold increase of the frequency of deaminase-induced Can^r^ clones (1.4 × 10^-4^) in strains with PmCDA1 (and a dramatic 15-fold increase in comparison to strains with vector alone, 9.3 × 10^-6^), but the frequency did not further increase on days 2–6, after growth cessation. Diploid strains were different. No canavanine-resistant clones were observed in diploids with vector alone at any day or with PmCDA1 vector on day 0, as expected from the low probability of the coincidence of any of the two rare spontaneous genetic events (**Figure 1**), as observed in our previous work (Lada et al., 2013). The frequency of the Can^r^ clones induced by deaminase expression on day 1 was 1.04 × 10^-6^ for *ung1* strains, just two orders of magnitude lower than for the haploid (much higher than theoretically expected 10^-8^) and 1.65 × 10^-7^ for the *UNG1* strain. It continued to increase steadily on days 3–6 (red boxes; differences are significant with *p* < 0.05 by one-way ANOVA with Bonferroni’s correction for all pairwise combinations of days, except for days 1 versus 2, 2 versus 3, and 4 versus 5) in apparently non-growing cultures (**Figure 2**, blue boxes). It is noteworthy that both *ung1* and *UNG1* diploid strains show the same trend, though the relative frequency of mutants is expectedly lower in the wild-type strain capable of repairing the uracil in DNA. This ruled out that the cause of mutant frequency raise was recombination induced by PmCDA1 in the Ung1^+^ diploids. APOBECs do not induce recombination in Ung1^-^ strains [see section “Introduction” and papers on recombination by AID (Di Noia and Neuberger, 2004; Poltoratsky et al., 2004) and PmCDA1 (Rogozin et al., 2007)] but the raise was observed in both diploid strains regardless of their UNG1 status.

### Mutation Loads in Genomes of Can^r^ Mutants Induced in Diploids on Different Days Are Similar

**FIGURE 3:**
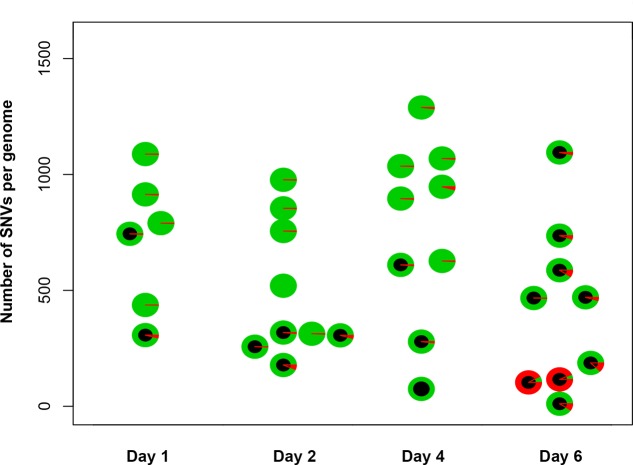
Genomes of Can^r^ mutants induced by PmCDA1 in diploids on different days possess a similar number of mutations. *Y*-axis, total number of SNVs per genome of mutant clones. Each circle on the plot represents an individual CAN^R^ or FOA^R^ mutant, and is colored according to the proportion of heterozygous (green sectors) and homozygous (red sectors) SNVs. The black circles are used to indicate the clones with a homozygous state of the mutant reporter gene (*CAN1* or *URA3*).

**FIGURE 4:**
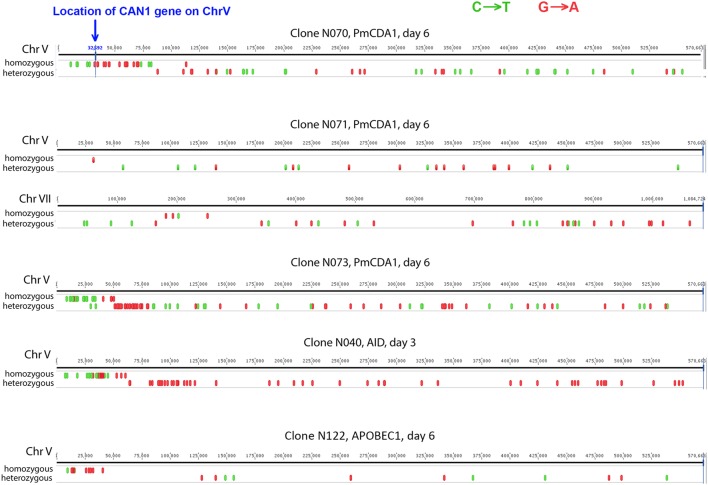
Characteristic mutation clusters associated with canavanine-resistant mutants induced by deaminases in diploids. Clusters of mutations induced by deaminases in several mutants. Individual chromosomes were visualized using Geneious (Biomatters).

To understand the mechanisms leading to the increase of the frequency of mutant clones appeared on different days of incubation in galactose-containing medium in resting diploids, we sequenced whole genomes of PmCDA1-induced *can1* and *ura3* mutants (the frequency of 5-FOA resistant mutants followed the same kinetics as Can^r^ mutants) obtained in the LAN210 strain. A summary of the results for the mutants induced by PmCDA1 is presented in **Figure 3** and detailed information is included in Supplementary Table 1, upper part. If deaminase kept inducing mutations in resting cells, where transcription was still ongoing with generally the same profile (see overall comparison of transcription profiles in Supplementary Figure 1 and, more specific, expression data for *PmCDA1*, *CAN1* and several housekeeping genes in Supplementary Table 2), we expect to see the time-dependent increase in the total number of mutations. Contrary to this expectation, the genomes of Can^r^ clones from days 1, 2, 4, and 6 had essentially the same loads of mainly C to T transition mutations, the type of mutations expected from cytosine deamination in the *ung1* strain (Lada et al., 2013) [Supplementary Table 1; Kruskal–Wallis test, *p* = 0.171, pairwise comparisons between groups of clones from different days (1, 2, 4, and 6) by Dunn’s multiple comparisons test are also non-significant]. This meant that most of the PmCDA1-induced mutations occurred before cell division stopped and the increase was mediated via one of the LOH mechanisms (**Figure 1**, left branch), even though *PmCDA1* was expressed on both day 3 and 6, as judged by RNA sequencing and thus could in theory induce transcription-dependent mutations in the *CAN1* gene and in the vicinity (black dots and red sectors, respectively, in **Figure 3**). Examples of such clones are presented in **Figure 4**, where in most of them homozygous mutations were found distally to the *CAN1* gene. APOBECs are known to induce predominantly heteroallelic mutations in reporter genes and heterozygous mutations elsewhere in the genome (even at the most prominent hotspots) in diploid *ung1* yeast strains (Lada et al., 2012; Taylor et al., 2014). Thus, we considered any enrichment in homozygous SNVs in the left arm of ChrV, where both *CAN1* and *URA3* genes reside, as clusters resulting from LOH. Some clones possessed only one SNV in left arm of ChrV (Supplementary Table 1, row K, and Supplementary Table 3), and this was classified as a LOH based on the above logic. We will describe the properties of the clusters in more depth later, but it was clear that recombination contributed to the increased frequency of recovery of Can^r^ clones on later days: more than half of clones from days 1, 2, and 4 does not possess neither homozygous mutations in *CAN1* nor homozygous mutation clusters (**Figure 3**). It is imperative to mention that recombination was induced genome wide, as exemplified by, e.g., clone N071, where products of recombination are also evident in chromosome VII (**Figure 4**). Because the increases in mutant clones in our work were seen both in *UNG1* and *ung1* strains (previous section), we are confident that the induction of recombination was not caused by deaminases. Therefore, we looked for the cause of induction of recombination in our experiments. AID has been shown to be able to substitute Spo11 in induction of DSB during meiotic recombination, but this effect was completely dependent on Ung1 (Pauklin et al., 2009). We questioned, whether the effects that we observed are due to Ung1-independent induction of recombination by deaminase, or a starvation-induced phenomenon?

### Recombination Is Induced in Starving Cells

**FIGURE 5:**
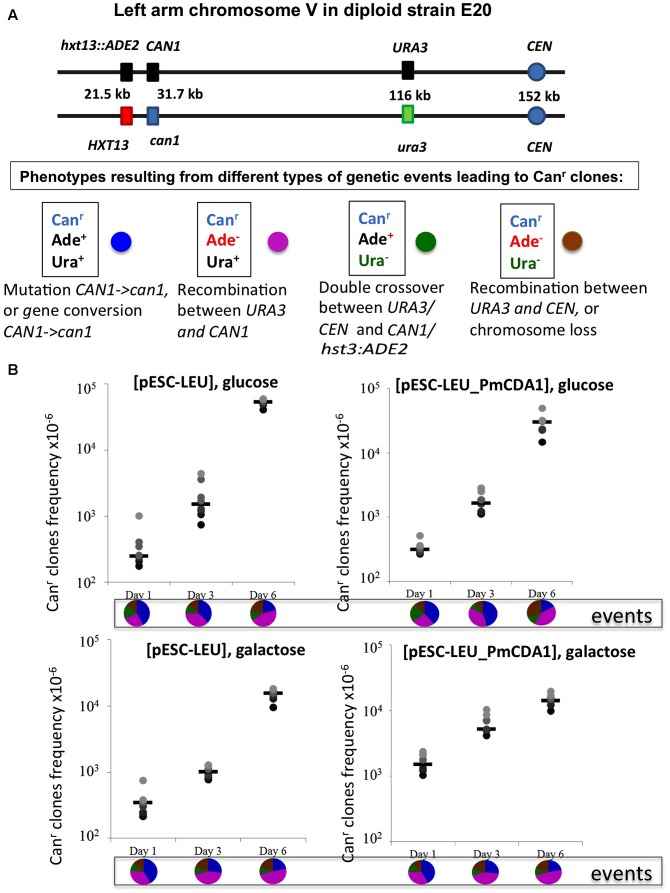
Recombination is induced by starvation in yeast diploids. **(A)** Chromosome V of the yeast diploid designed to study the frequency and types of recombination events leading to canavanine-resistant clones is drawn in upper part. The distances between the left end of the chromosome and each genetic marker are shown. Phenotypes resulting from different classes of genetic events are shown below. Each phenotype received a color code represented by a circle. **(B)** Graphs illustrating the increase of the frequency of Can^r^ mutants during prolonged incubation of yeast in glucose or galactose in strains with or without deaminase. Below are pie charts representing a proportion of different genetic events on different days by color codes assigned in panel **(A)**.

We constructed an *ung1* diploid heterozygous for the *CAN1* gene and additional markers on chromosome V, which allows for classification of recombination events on the left arm of the chromosome (see section “Materials and Methods” and **Figure 5A**). This strain is canavanine-sensitive due to the presence of one copy of the wild-type *CAN1* gene. The frequency of canavanine-resistant clones on day 1 with the vector alone was already high, and similar in glucose and galactose-containing media (from 2 to 6 × 10^-4^, **Figure 5B**), as expected from the known high frequency of mitotic recombination in yeast generally exceeding the rate of spontaneous mutations by orders of magnitude (Kunz and Haynes, 1981). We have found that the frequency of these resistant clones further increases on day 3 and on day 6 reaching the range with 10^-2^, the two orders of magnitude increase over day 1. The rate of accumulation of Can^r^ clones in cultures with PmCDA1 in glucose medium is essentially the same. When the strain was incubated in galactose, the frequency of resistant colonies on days 1 and 3 is almost 10-fold higher in comparison to glucose cultures, as expected from a potent mutagenic effect of PmCDA1 (10^-4^, previous section), but the final frequency on day 6 was like other variants. We analyzed these colonies as described in section “Materials and Methods.” The results are in pie diagrams below each dataset in graphs in **Figure 5B**. The predominant class of events leading to canavanine-resistant clones on day 1 was mutations in *CAN1* or gene conversion but on day 6 this class was shrunk and recombination in the long 80 kb interval between *URA3* and *CAN1* became a prevailing class (**Figure 5B**, *p*-values are in Supplementary Table 4). To distinguish between gene conversion and a second (independent) mutation, we sequenced *CAN1* genes from several resistant clones. In the strains with a control vector, 18 out of 20 variants had homozygous original mutation, G1018A (i.e., gene conversion becomes the main factor contributing to their appearance). At conditions when PmCDA1 was active, only 4 out of 21 clones were homozygous, whereas other clones had an additional single mutation elsewhere in the *CAN1* gene (Supplementary Table 5). This explains the elevated frequency of canavanine-resistant clones on day 1 in strains with PmCDA1 grown in galactose.

### Mechanisms of Mutation Induction in Diploids by Lamprey PmCDA1 and Mammalian Deaminases AID and APOBEC1 Are Similar

We have also sequenced the genomes of mutants induced by human deaminase AID and rat enzyme APOBEC1 at days 1, 3, and 6 (Supplementary Table 1, lower part). We found similar clusters of homozygous and heterozygous mutations near the reporter locus (Supplementary Table 3). We concluded that all deaminases induced resistant clones in resting cells by a similar mechanism.

### Starvation Leads to Meiotic-Like Levels of Recombination and Meiosis at Late Stages of the Stationary Phase

There is an increase in the number of homozygous SNVs in clones induced on day 6 of growth, for both PmCDA1 and other deaminases (**Figure 3** and Supplementary Table 1). Upon visual examination in the genome browser, it turned out that many of these clones possess strong clusters of homozygous SNVs on the left arm of chromosome V, where the reporter gene *CAN1* is located (illustrated in **Figure 4**). Same was seen for *URA3* for some of the clones (see Supplementary Table 1). Clearly, clones with recombination-generated homozygous clusters distal to the *CAN1* were predominant on the day 6. Moreover, some of the clones from day 6 possessed additional clusters of homozygous SNVs on other chromosomes (Supplementary Table 3). It is interesting that part of clusters distal to the reporter gene were frequently homozygous and those parts of clusters which were proximal to the reporter gene were heterozygous (representative recombinational clusters are shown on **Figure 4** and Supplementary Figure 2). In some cases, crossing-over led to homozygous *can1* distal clustered mutations and proximal mutations were represented by reciprocal changes, C to T and G to A, indicating that they might originate from deamination of opposite recessed DNA strands from both sides of double strand break, as seen in (Taylor et al., 2013).

### Cessation of Growth and Long Starvation of Diploids Leads to Appearance of Haploids

We have found several clones on day 6 with a relatively low number of homozygous mutations in their genomes. Some of them had an “a” or “alpha” mating type, so they were presumably haploids (Supplementary Table 1, row J). Apparently, starvation in our experiments leads to the start of induction of meiotic program that in a few cases was completed. The colonies consisting of haploid cells out of the four-spore asci with the two Can^+^ and two Can^-^ spores will arise quite frequently (25%) if canavanine kills cells prior in ascus before they mate, so *can1* cells will have the same mating type and will grow as haploids. Also, the high lethality of haploids with numerous mutations (Lada et al., 2013) may contribute to the recovery of isolated haploid descendants out of the four-spore asci. Obviously, haploid clones may have been descendant of regular, not hypermutable, cell fraction and had much less mutations in their genomes.

## Discussion

We described the phenomenon of increased recovery of drug-resistant mutants induced by APOBECs throughout the duration of the stationary growth phase in non-dividing diploid yeast cells. Whole-genome sequencing, combined with classical yeast genetics, has shown that the predominant mechanism is the LOH, with an additional minor component related to APOBECs action during recombination. In yeast system, increase of mitotic recombination happens because of the induction of commitment to meiosis with a characteristic high meiotic-like recombination rates but with mitotic chromosome segregation (Esposito and Esposito, 1974) and eventually meiosis itself. Recently, this process, referred as to return to growth, have been characterized using whole-genome sequencing (Laureau et al., 2016). The mechanism may have implications for understanding the action of APOBECs in carcinogenesis (Lada et al., 2015).

Mutagenesis is fundamental for evolution, including the evolution of tumors. The classical paradigm of random mutation generation in growing cells (Luria and Delbruck, 1943) was first challenged in the 1970s, when it has been shown that mutations can be induced in non-dividing cells (Eckardt and Haynes, 1977). In the 1980s, this was developed further with the discovery of adaptive mutations (Shapiro, 1984; Cairns et al., 1988; Rosenberg, 2001). More recently, with the emergence of whole-genome sequencing, it has been extensively shown that mutations are distributed in the genome in a highly non-random fashion and, in some regions, mutations occur in clusters. The mechanisms of mutations in non-growing cells are related to ongoing transcription and DNA repair processes.

**FIGURE 6:**
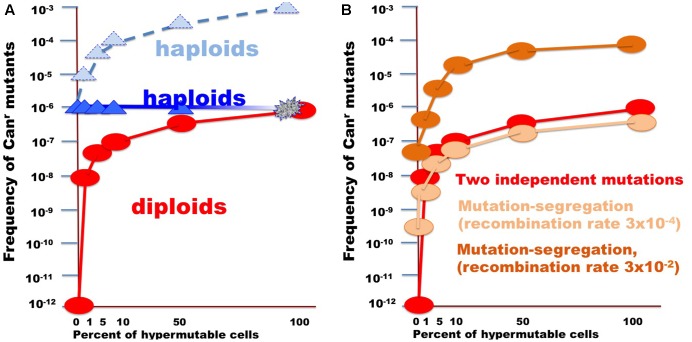
Illustration of the effect of the size of fraction of hypermutable cells on the apparent mutation rate in diploids versus haploids. **(A)** For the example, the *CAN1* mutation rate was assumed to be 10^-6^, thus two copies in diploids will simultaneously mutate at a rate of 10^-12^. Mutation rate in hypermutable cells was set to 10^-3^. Such a rate is incompatible with life in haploids. Therefore, despite the predicted raise of mutation frequency (light blue triangles connected by the dashed blue line), the actual frequency stays constant (blue triangles) until the fraction of the hypermutable cells approaches 100% and cells die (illustrated by fading blue line). Hypermutable cells are viable in diploids. The initial appearance of hypermutable cells in diploids leads to four orders of magnitude rise of mutant recovery due to coincident mutation in the two homologs (**Figure 1**) and with the growth of the fraction the frequency of mutants get closer to the frequency in haploids (red circles and line). **(B)** Curves illustrating comparative frequency of resistant mutant in diploids with different size of hypermutable fraction via coincidence of mutations (red circles and lines, same as in **A**) versus mutation-segregation mechanism (light brown, recombination frequency 3 × 10^-4^; dark brown color, recombination frequency 3 × 10^-4^).

In the current work, we analyzed factors that influence the distribution of mutations induced by DNA deamination in non-dividing diploid yeast cells. We have expressed AID/APOBEC cytosine deaminase PmCDA1 in diploid yeast strains and observed an increase in the recovery of drug-resistant mutant clones after the cells reached the stationary phase (**Figure 2**). We have sequenced genomes of mutants induced by PmCDA1, as well as by AID and APOBEC1, in the early and late stationary phase (days 2, 4, and 6 after the culture start for PmCDA1, and days 3 and 6 after the culture start for AID and APOBEC1, see **Figure 3**), and found hundreds to thousands of passenger SNVs (Supplementary Table 1 and Supplementary Data Sheet 1; Lada et al., 2013). Surprisingly, we have found no difference in the number of mutations in the genomes of early and late clones. The explanation for these observations is presented in **Figure 6**. According to the concept of a hypermutable fraction of cells, in growing cells (Lada et al., 2013), APOBECs induced mutation rates are uneven among cells. The illustration in **Figure 6A**, shows that, although, in theory, haploids and diploids have the same dependence of mutant frequency on the size of the hypermutable fraction of cells (compare pale blue and red lines), we never see the contribution of these cells to mutation rates in haploids, because haploid cells have a certain threshold for tolerable mutation rates (Herr et al., 2011), thus most mutable cells die in haploids (fading blue line ending with a mark symbolizing cells extinction), lowering the observed mutation frequency (left part of the straight blue line). As a result, the observed frequency of drug-resistant mutants in diploids is higher than expected, in extreme reaching the level of mutants in haploids. **Figure 6B** illustrates how the frequency of recovered mutants in diploids is affected by the rate of LOH. For example, if the frequency of recombination is low, we will mostly recover mutants induced by the “mutation coincidence mechanism” (compare the dark brown curve with the red curve). This is what we found in our earlier work with base analog and PmCDA1 (Lada et al., 2013) and at days 1–4 in the current study. If recombination frequency is increased 100-fold, we will recover the mutants, which occurred by the two mechanisms, at the same proportion (light brown and red lines) and if recombination is higher than mutation frequency, we will mostly see clones with LOH, like we saw at day 6 of starvation. Therefore, this additional mechanism of mutant generation will add to the recovered frequency of drug-resistant mutants. In our work, this mechanism led to the appearance of clusters of homozygous mutations distal to the site of the reporter gene. The high frequency of the recovery of such mutants will still depend on the transiently hypermutable cells that experienced most DNA damage by APOBECs. The genomes of resistant clones the mutation loads will be the same at different days. Most Can^r^ clones in heterozygous diploid represented recombination events in the region between the *URA3* and *CAN1* genes (**Figure 5B**), reflecting the longest distance between these markers.

**FIGURE 7:**
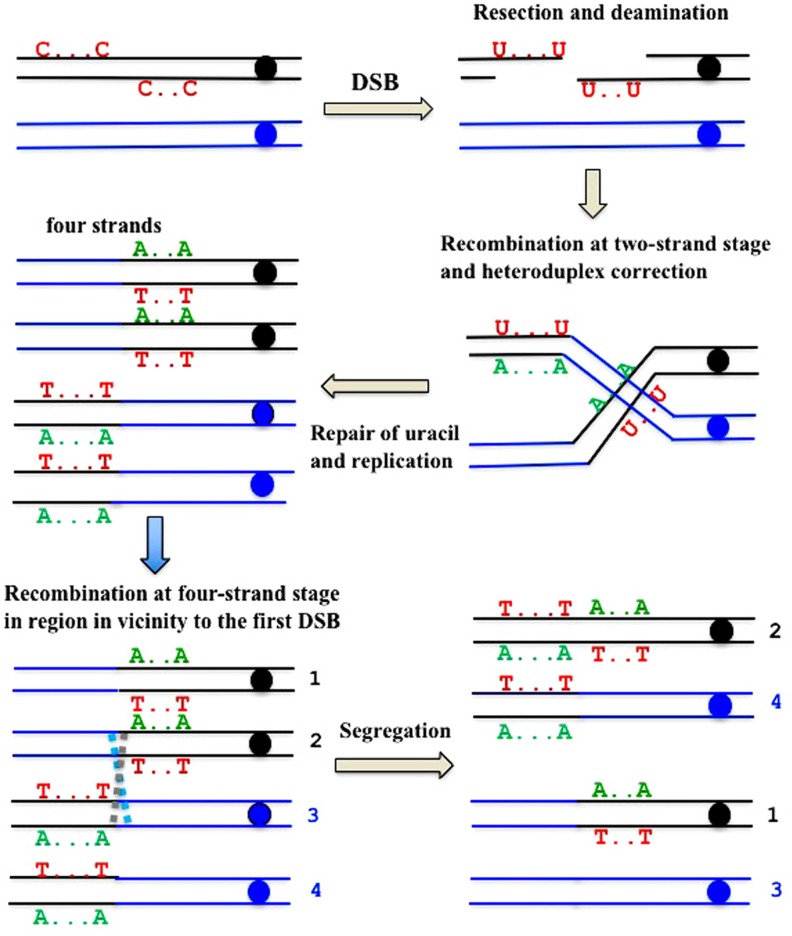
Formal explanation of the mechanism of the formation of deaminase-induced homo-heterozygous mutation clusters with reciprocal polarity of transitions. Double strand break ends are resected and exposed single-stranded regions are deaminated. Deaminated “C”s are shown only in the original parent molecule. Recombination at the two-stand stage and the subsequent repair of heteroduplexes and uracil is followed by recombination at the four-strand stage in the site close to the first exchange.

There is a high proportion of strong clusters of homozygous SNVs in the genomes of mutants induced by deaminases selected on the sixth day of growth. It confirms that recombination contributes to the induction of these mutants. Interestingly, some of the clones possess long clusters, which are rich in homozygous mutations, whereas others contain only several (sometimes single) homozygous mutations. We classify the clones with single homozygous SNV in the reporter gene as recombination-induced, because normally APOBEC-induced homozygous mutations can be found only at the strongest mutational hotspots, where both chromosomes are likely to be independently targeted (Lada et al., 2012). In both cases, the “homozygous” clusters also usually contained additional heterozygous mutations. It suggests that APOBEC deaminates resected ssDNA during recombination, leading to clusters of homozygous mutations. Then, additional deamination can happen on the ssDNA generated via various DNA transactions, resulting in heterozygous mutations within the cluster, which originally appeared due to recombination (**Figure 7**).

Based on these and other results, we propose the following model of generation of drug-resistant mutant clones in the non-dividing diploid cells. Mutations are generated genome-wide or locally in the presence of an ssDNA-specific mutagen, deaminase. Their induction occurs during active replication and transcription because of the many regions of transiently unprotected ssDNA, resulting in single and clustered mutations (Taylor et al., 2014; Lada et al., 2015; Bhagwat et al., 2016; Haradhvala et al., 2016; Hoopes et al., 2016; Seplyarskiy et al., 2016). We have seen the accumulation of genomic mutations only in actively dividing cells, and there was no further accumulation without replication. We explain the absence of a transcriptional component of APOBEC-induced deamination in the resting cell as following. Even if pre-mutational states (U:G mismatch in this case) were still induced in resting cells during ongoing transcription, they would never have a chance to be fixed as complete mutations, because mutation fixation (at least in our system) depends on replication, but cells cannot replicate in the presence of canavanine. Induction of recombination in stationary cells led to the LOH, thus leading to a boost for the recovery of drug-resistant mutant clones. Two processes—the loss of protection of ssDNA during replication/transcription/recombination, and deamination—act synergistically to shape the mutational profiles in the genomes of mutant cells. The hidden burden of heterozygous mutations can be exposed by recombination and other LOH evens in resting cells, explaining sudden changes of cell’s characteristics. These processes in mammalian dormant cells may lead to oncogenic transformation.

## Author Contributions

AL and YP incepted the study. AL, SK, and IR designed and performed NGS data analysis. AL did yeast mutagenesis studies. ES did recombination experiments. AD and DP executed NGS. AL, ES, IR, AZ, and YP analyzed and interpreted the results. AL, ES, and YP wrote the manuscript that was edited and approved by all authors.

## Conflict of Interest Statement

The authors declare that the research was conducted in the absence of any commercial or financial relationships that could be construed as a potential conflict of interest.
