# Supplementary material for: Recombination Is Responsible for the Increased Recovery of Drug-Resistant Mutants with Hypermutated Genomes in Resting Yeast Diploids Expressing APOBEC Deaminases
Source: Front Genet. 2017 Dec 12;8:202. doi: 10.3389/fgene.2017.00202 (PMC5733079; doi:10.3389/fgene.2017.00202)
Supplement: Supplementary file 4 [file Table_4.docx]

**Supplemental Table 4.**

Fisher's exact test validating that a proportion of mutation/conversion events **(Fig 5B)**decreased and a proportion of recombination events increased over time in starving cells. Note the drastic difference between Day 1 and Day 6 (values are in bold).

| Strain | Media | P-value | | |
| --- | --- | --- | --- | --- |
|  |  | Day1 vs Day3 | **Day1 vs Day6** | Day3 vs Day6 |
| ES20 [pESC-LEU] | GLU | P=0.0002 | **P<0.0001** | P=0.0488 |
|  | GAL | P=1 | **P<0.0001** | P<0.0001 |
| ES20 [pESC-PmCDA1] | GLU | P=0.7 | **P<0.0001** | P<0.0001 |
|  | GAL | P=0.5 | **P<0.0001** | P=0.0201 |
