# Supplementary material for: Recombination Is Responsible for the Increased Recovery of Drug-Resistant Mutants with Hypermutated Genomes in Resting Yeast Diploids Expressing APOBEC Deaminases
Source: Front Genet. 2017 Dec 12;8:202. doi: 10.3389/fgene.2017.00202 (PMC5733079; doi:10.3389/fgene.2017.00202)
Supplement: Supplementary file 5 [file Table_5.docx]

**Supplemental Table 5. Sequence changes in *CAN1* gene in Can^+^Ade^+^ Ura^+^ clones induced by PmCDA1*.**

| Number of mutant | Additional heterozygous mutations | Additional homozygous mutations |
| --- | --- | --- |
| 2,12,17,2-2 | none |  |
| 5 | 91 C->T |  |
| 18 | 452 C->T |  |
| 16 | 577 C->T |  |
| 19 | none | 612 G->A, 627 G->A |
| 8 | 656 C->T |  |
| 20 | 673 C->T |  |
| 13,14 | 1163 C->T |  |
| 8-2 | 1175 C->T |  |
| 11 | 1195 C->T |  |
| 4,6,7,3-2 | 1214 C->T |  |
| 3 | 1221 C->T |  |
| 1 | 1535 Т->G |  |
| 10 | 1537 C->T |  |
| Total=21 clones | | |

* Analysis was done for Can^r^ clones arising after one day of incubation of ES20 with PmCDA1 in galactose. In 4 (20%) the preexisting heterozygous mutation *can1* 1018G->A became homozygous (first row). In the other clones the original mutation was remained heterozygous and the clone acquired additional heterozygous mutations listed in column two. One clone possessed two additional homozygous mutations (column 3).

For comparison, we also sequenced 20 spontaneous Can^r^ clones of ES20 with empty vector selected under same conditions. In 18 (90%) the preexisting heterozygous mutation *can1* 1018G->A became homozygous. In two clones the original mutation stayed heterozygous and additional heterozygous mutations, 311С->T in one clone and 1163C->T in another, were present.
