# Supplementary figures and images for: Recombination Is Responsible for the Increased Recovery of Drug-Resistant Mutants with Hypermutated Genomes in Resting Yeast Diploids Expressing APOBEC Deaminases
Source: Front Genet. 2017 Dec 12;8:202. doi: 10.3389/fgene.2017.00202 (PMC5733079; doi:10.3389/fgene.2017.00202)

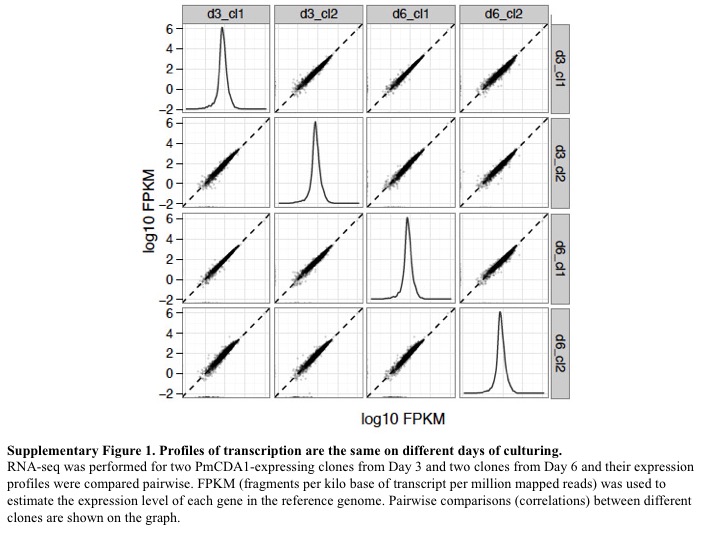

Supplement: Supplementary file 7 [file Image_1.JPEG]

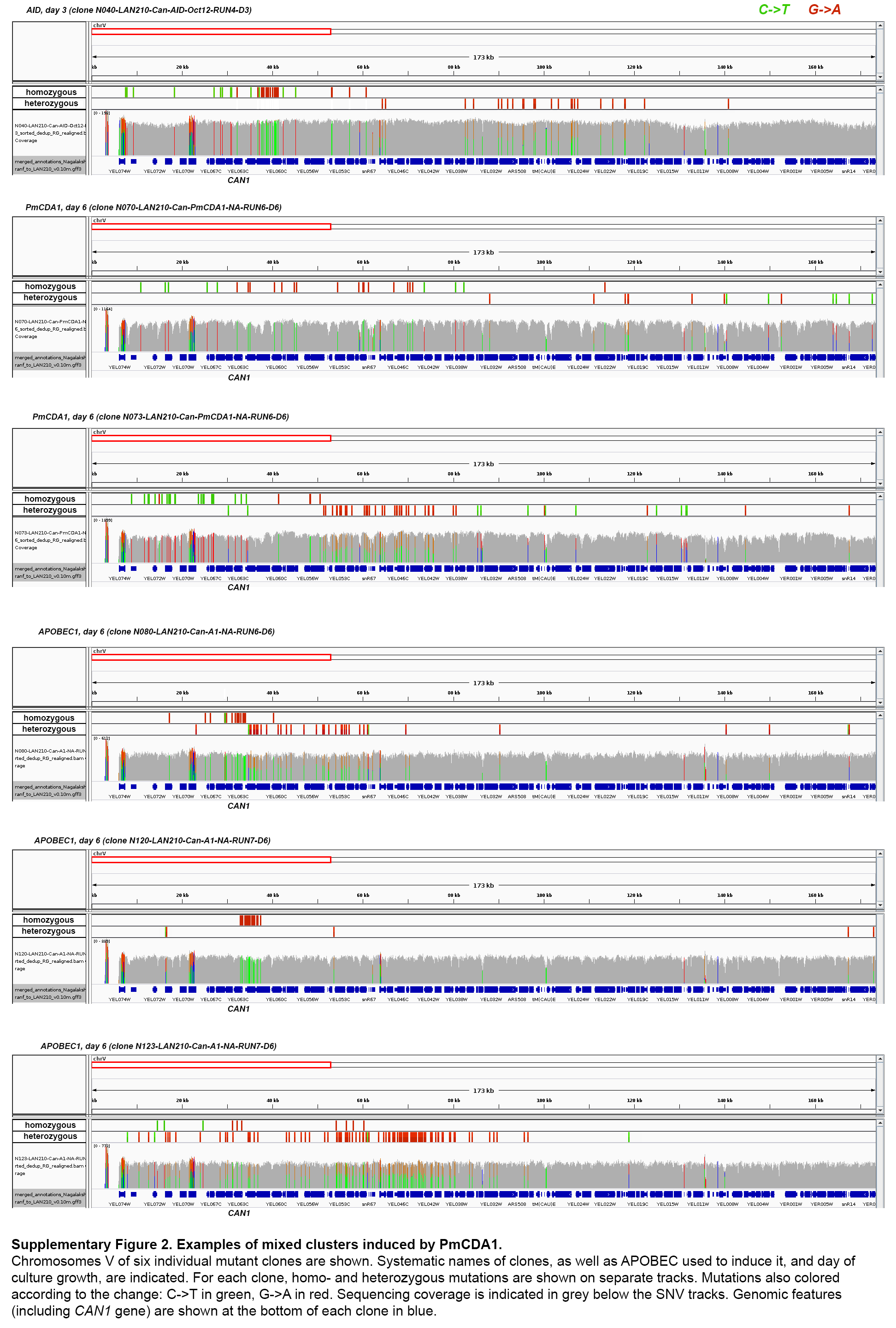

Supplement: Supplementary file 8 [file Image_2.tif]
